# Associations between near end-of-life flortaucipir PET and postmortem CTE-related tau neuropathology in six former American football players

**DOI:** 10.1007/s00259-022-05963-x

**Published:** 2022-09-24

**Authors:** Michael L. Alosco, Yi Su, Thor D. Stein, Hillary Protas, Jonathan D. Cherry, Charles H. Adler, Laura J. Balcer, Charles Bernick, Surya Vamsi Pulukuri, Bobak Abdolmohammadi, Michael J. Coleman, Joseph N. Palmisano, Yorghos Tripodis, Jesse Mez, Gil D. Rabinovici, Kenneth L. Marek, Thomas G. Beach, Keith A. Johnson, Bertrand Russell Huber, Inga Koerte, Alexander P. Lin, Sylvain Bouix, Jeffrey L. Cummings, Martha E. Shenton, Eric M. Reiman, Ann C. McKee, Robert A. Stern, for the DIAGNOSE C. T. E. Research Project, Eric Reiman, Yi Su, Kewei Chen, Hillary Protas, Connie Boker, Michael L. Alosco, Rhoda Au, Robert C. Cantu, Lindsay Farrer, Robert Helm, Douglas I. Katz, Neil Kowall, Jesse Mez, Gustavo Mercier, James Otis, Robert A. Stern, Jason Weller, Irene Simkin, Alondra Andino, Shannon Conneely, Courtney Diamond, Tessa Fagle, Olivia Haller, Tennyson Hunt, Nicole Gullotti, Megan Mariani, Brian Mayville, Kathleen McLaughlin, Mary Nanna, Taylor Platt, Surya Pulukuri, Fiona Rice, Madison Sestak, Michael McClean, Yorghos Tripodis, Douglas Annis, Christine Chaisson, Diane B. Dixon, Carolyn Finney, Kerrin Gallagher, Kaitlin Hartlage, Jun Lu, Brett Martin, Emmanuel Ojo, Joseph N. Palmisano, Brittany Pine, Janani Ramachandran, Sylvain Bouix, Jennifer Fitzsimmons, Alexander P. Lin, Inga K. Koerte, Ofer Pasternak, Martha E. Shenton, Hector Arcinieago, Tashrif Billah, Elena Bonke, Katherine Breedlove, Eduardo Coello, Michael J. Coleman, Leonhard Jung, Huijun Liao, Maria Loy, Elizabeth Rizzoni, Vivian Schultz, Annelise Silva, Brynn Vessey, Tim L. T. Wiegand, Sarah Banks, Charles Bernick, Jason Miller, Aaron Ritter, Marwan Sabbagh, Raelynn de la Cruz, Jan Durant, Morgan Golceker, Nicolette Harmon, Kaeson Kaylegian, Rachelle Long, Christin Nance, Priscilla Sandoval, Robert W. Turner, Kenneth L. Marek, Andrew Serrano, Charles H. Adler, David W. Dodick, Yonas Geda, Jennifer V. Wethe, Bryce Falk, Amy Duffy, Marci Howard, Michelle Montague, Thomas Osgood, Debra Babcock, Patrick Bellgowan, Laura Balcer, William Barr, Judith Goldberg, Thomas Wisniewski, Ivan Kirov, Yvonne Lui, Charles Marmar, Lisena Hasanaj, Liliana Serrano, Alhassan Al-Kharafi, Allan George, Sammie Martin, Edward Riley, William Runge, Jeffrey L. Cummings, Elaine R. Peskind, Elizabeth Colasurdo, Daniel S. Marcus, Jenny Gurney, Richard Greenwald, Keith A. Johnson

**Affiliations:** 1 Boston University Alzheimer’s Disease Research Center, Boston University CTE Center, Department of Neurology, Boston University School of Medicine, Boston, MA USA; 2 Banner Alzheimer’s Institute, Arizona State University, and Arizona Alzheimer’s Consortium, Phoenix, AZ USA; 3 VA Boston Healthcare System, Boston, MA USA; 4 Framingham Heart Study, Framingham, MA USA; 5 VA Bedford Healthcare System, Bedford, MA USA; 6 Banner Alzheimer’s Institute, Arizona Alzheimer’s Consortium, Phoenix, AZ USA; 7 Department of Neurology, Mayo Clinic College of Medicine, Mayo Clinic Arizona, Scottsdale, AZ USA; 8 Departments of Neurology, Population Health and Ophthalmology, NYU Grossman School of Medicine, New York, NY USA; 9 Cleveland Clinic Lou Ruvo Center for Brain Health, Las Vegas, NV USA; 10 Department of Neurology, University of Washington, Seattle, WA USA; 11 Psychiatry Neuroimaging Laboratory, Department of Psychiatry, Brigham and Women’s Hospital, Boston, MA USA; 12 Biostatistics and Epidemiology Data Analytics Center (BEDAC), Boston University School of Public Health, Boston, MA USA; 13 Department of Biostatistics, Boston University School of Public Health, Boston, MA USA; 14 Memory & Aging Center, Departments of Neurology, Radiology & Biomedical Imaging, University of California San Francisco, San Francisco, CA USA; 15 Institute for Neurodegenerative Disorders, Invicro, LLC, New Haven, CT USA; 16 Banner Sun Health Research Institute, Sun City, Arizona USA; 17 Massachusetts General Hospital, Boston, MA USA; 18 Harvard Medical School, Boston, MA USA; 19 Gordon Center for Medical Imaging, Boston, MA USA; 20 Brigham and Women’s Hospital, Boston, MA USA; 21 National Center for PTSD, VA Boston Healthcare, Jamaica Plain, MA USA; 22 cBRAIN, Department of Child and Adolescent Psychiatry, Psychosomatics and Psychotherapy, Ludwig Maximilians University, Munich, Germany; 23 Graduate School of Systemic Neurosciences, Ludwig Maximilians University, Munich, Germany; 24 NICUM (NeuroImaging Core Unit Munich), Ludwig Maximilians University, Munich, Germany; 25 Center for Clinical Spectroscopy, Department of Radiology, Brigham and Women’s Hospital, Harvard Medical School, Boston, MA USA; 26 Chambers-Grundy Center for Transformative Neuroscience, Department of Brain Health, School of Integrated Health Sciences, University of Nevada Las Vegas, Las Vegas, NV USA; 27 Department of Radiology, Brigham and Women’s Hospital, Harvard Medical School, Boston, MA USA; 28 Banner Alzheimer’s Institute, University of Arizona, Arizona State University, Translational Genomics Research Institute, and Arizona Alzheimer’s Consortium, Phoenix, AZ USA; 29 Departments of Neurosurgery, and Anatomy & Neurobiology, Boston University School of Medicine, Boston, MA USA

**Keywords:** Biomarkers, Chronic traumatic encephalopathy, Football, Neurodegenerative disease, Positron emission tomography imaging, Repetitive head impacts, Tau, Flortaucipir

## Abstract

**Purpose:**

Flourine-18-flortaucipir tau positron emission tomography (PET) was developed for the detection for Alzheimer’s disease. Human imaging studies have begun to investigate its use in chronic traumatic encephalopathy (CTE). Flortaucipir-PET to autopsy correlation studies in CTE are needed for diagnostic validation. We examined the association between end-of-life flortaucipir PET and postmortem neuropathological measurements of CTE-related tau in six former American football players.

**Methods:**

Three former National Football League players and three former college football players who were part of the DIAGNOSE CTE Research Project died and agreed to have their brains donated. The six players had flortaucipir (tau) and florbetapir (amyloid) PET prior to death. All brains from the deceased participants were neuropathologically evaluated for the presence of CTE. On average, the participants were 59.0 (SD = 9.32) years of age at time of PET. PET scans were acquired 20.33 (SD = 13.08) months before their death. Using Spearman correlation analyses, we compared flortaucipir standard uptake value ratios (SUVRs) to digital slide-based AT8 phosphorylated tau (p-tau) density in a priori selected composite cortical, composite limbic, and thalamic regions-of-interest (ROIs).

**Results:**

Four brain donors had autopsy-confirmed CTE, all with high stage disease (*n* = 3 stage III, *n* = 1 stage IV). Three of these four met criteria for the clinical syndrome of CTE, known as traumatic encephalopathy syndrome (TES). Two did not have CTE at autopsy and one of these met criteria for TES. Concomitant pathology was only present in one of the non-CTE cases (Lewy body) and one of the CTE cases (motor neuron disease). There was a strong association between flortaucipir SUVRs and p-tau density in the composite cortical (*ρ* = 0.71) and limbic (*ρ* = 0.77) ROIs. Although there was a strong association in the thalamic ROI (*ρ* = 0.83), this is a region with known off-target binding. SUVRs were modest and CTE and non-CTE cases had overlapping SUVRs and discordant p-tau density for some regions.

**Conclusions:**

Flortaucipir-PET could be useful for detecting high stage CTE neuropathology, but specificity to CTE p-tau is uncertain. Off-target flortaucipir binding in the hippocampus and thalamus complicates interpretation of these associations. In vivo biomarkers that can detect the specific p-tau of CTE across the disease continuum are needed.

## Introduction

Chronic traumatic encephalopathy (CTE) is a neurodegenerative disease that has been diagnosed in the postmortem brains of individuals exposed to repetitive head impacts (RHI), particularly former American football players [1–5]. A diagnosis of CTE can be made only by neuropathological examination that shows phosphorylated tau (p-tau) in neurons around small blood vessels at the depths of the sulci [6, 7]. Aggregation of p-tau epicenters begins in the frontotemporal cortices [7, 8]. Medial temporal lobes (MTL) are typically affected in later disease stages [7, 8]. Like Alzheimer’s disease (AD), the tau aggregates of CTE consist of mixed three (3R) and four (4R) microtubule binding site repeat motifs. However, evidence shows a shift from 4 to 3R in the tau aggregates with disease progression in CTE [9]. The molecular composition of p-tau in CTE is also unique from AD and frontotemporal lobar degeneration (FTLD), as is the distribution of the tau tangle pathology [10–12]. Unlike aging-related tau astrogliopathy (ARTAG), the p-tau lesion in CTE must be neuronal [13]. Neuritic amyloid plaques are not diagnostic and often absent in CTE [5, 7, 14].

Validation of in vivo biomarkers for the detection of the p-tau in CTE does not yet exist, thereby contributing to the inability to accurately diagnose CTE during life [15]. Advances have been made in the identification of biomarkers of CTE [16–19]. Positron emission tomography (PET) has the potential to characterize the tau tangle changes in CTE. Case studies have examined the usefulness of the tau tracer floruine-18-FDDNP for the detection of CTE [20–23]. However, the broader literature on this tracer shows it to have many limitations, including non-specific binding[24] and low signal-to-noise ratio [25]. Attention has shifted to the tau radioligand fluorine-18-flortaucipir [26–30]. This ligand detects the paired helical filament (PHF) tau in AD[31–33] and has been approved by the Food and Drug Administration (FDA) for this purpose. The value of flortaucipir to bind to tau aggregates in other tauopathies, including frontotemporal degenerative disorders[31, 34–38] and CTE, is less clear [29, 30]. This is perhaps due to the biochemical composition of tau in primary tauopathies, differences in molecular structure of the p-tau aggregates between the different tauopathies including between CTE and AD [10, 11], the spatial distribution and severity of tau pathology, as well as other reasons.

Stern et al.[29] observed higher mean flortaucipir SUVRs in the bilateral superior frontal cortices, bilateral MTL, and left parietal lobe among 26 symptomatic former National Football League (NFL) players (ages 40–69) compared with 31 same age asymptomatic men without a history of traumatic brain injury (TBI). Effect sizes were small and SUVRs were lower than those reported in AD [39]. Lesman-Segev et al.[30] observed a similar pattern among 11 men (10 former football players, ages 30 s to 70 s) diagnosed with the clinical syndrome of CTE known as traumatic encephalopathy syndrome (TES), based on the 2014 research diagnostic criteria [40]. The distribution of flortaucipir retention was consistent with high stage CTE with intense uptake in two TES participants who were amyloid positive and variable uptake among the nine amyloid-negative TES participants. Other data on flortaucipir-PET in CTE are from case reports [26, 28]. Autoradiography research has shown minimal flortaucipir binding in postmortem CTE tissue across the disease continuum [41]. The use of ethanol wash complicates the interpretation of the findings as has been alluded to in other autoradiography studies of flortaucipir [31].

PET to autopsy studies are needed to validate biomarkers using neuropathological standards [32, 42]. To date, there has only been a single case report of flortaucipir PET to autopsy study in CTE [27], a deceased former NFL player with autopsy-confirmed severe CTE who had a flortaucipir-PET 52 months prior to death. The predominant frontotemporal distribution of flortaucipir retention corresponded to CTE p-tau distribution at autopsy. There was a modest and non-significant correlation between flortaucipir SUVR and tau area fraction at autopsy. Larger flortaucipir-neuropathological correlation studies are needed to clarify these findings and to determine the usefulness of flortaucipir-PET for detecting CTE. In the present study, we examined the association between antemortem flortaucipir-PET uptake and postmortem p-tau neuropathology in six deceased former elite American football players.

## Materials and methods

### Participants and study design

The sample included six male former American football players (*n* = 3 former NFL players, *n* = 3 former college football players) who participated in the “Diagnostics, Imaging, and Genetics Network for the Objective Study and Evaluation of Chronic Traumatic Encephalopathy (DIAGNOSE CTE) Research Project” [43]. Participants underwent a 3-day baseline visit that consisted of neurological and neuropsychological examinations, MRI, and flortaucipir and florbetapir PET scans for tau and amyloid imaging, respectively. One participant in the current postmortem study did not have an MRI. All participants in the DIAGNOSE CTE Research Project were asked to donate their brain to the Veterans Affairs-Boston University-Concussion Legacy Foundation (VA-BU-CLF) brain bank and to become part of the Understanding Neurologic Injury in Traumatic Encephalopathy (UNITE) study [44]. The sample for the present study includes all former American football players from the DIAGNOSE CTE Research Project who died and donated their brains to the UNITE study as of March 2022. On average, the time from PET scans to death was 20.33 months (SD = 13.08, range = 4–41 months). Average postmortem interval was 49.17 (SD = 22.38) h. Reported causes of death included cancer, cardiovascular disease, falls, and motor neuron disease (Table 1). Four of the six former football players met criteria for TES, which was adjudicated while the participants were alive as part of DIAGNOSE CTE Research Project multidisciplinary diagnostic conferences and using the 2021 National Institute of Neurological Disorders and Stroke (NINDS) consensus diagnostic criteria [15]. TES was developed to represent the clinical syndrome associated with underlying CTE neuropathology [15]. The NINDS TES criteria include provisional levels of certainty for CTE pathology (i.e., suggestive of CTE, possible CTE, probable CTE). Specific criteria for the levels of certainty include clinical presentation, course, degree of RHI exposure, and level of functional impairment.

**Table 1:** Sample characteristics

	Case 1 (no CTE)	Case 2 (no CTE)	Case 3 (CTE stage III)	Case 4 (CTE stage III)	Case 5 (CTE stage III)	Case 6 (CTE stage IV)
Demographic and athletic characteristics						
Age	45–49	45–49	60–64	60–64	65–69	70–74
PET to death (months)	25	25	17	10	4	41
Sex	Male	Male	Male	Male	Male	Male
Racial identity	White	White	White	White	White	White
Level of play	College	College	NFL	College	NFL	NFL
Years of play	10	11	19	11	20	24
Clinical and genetic status						
Traumatic encephalopathy syndrome (TES)	No	Yes	No	Yes	Yes	Yes
TES-cognitive impairment	No	No	No	Yes	No	Yes
TES-neurobehavioral dysregulation	No	Yes	No	Yes	Yes	Yes
TES-dementia	No	No	No	Mild dementia	No	No
Level of certainty for CTE pathology	N/A	Suggestive	N/A	Probable	Suggestive	Possible
FAQ-Informant	0	1	0	13	5	5
FAQ-Participant	0	0	0	13	0	0
MoCA score	21	26	27	20	28	23
APOE status	ε2 / ε3	ε3 / ε3	Missing	ε3 / ε3	ε3 / ε3	ε3 / ε4
Florbetapir PET						
SUVR	0.97	0.94	1.00	0.93	Not done	1.08
Interpretation	Negative	Negative	Negative	Negative	Not done	Negative
Neuropathological diagnosis						
CTE	Absent	Absent	Present	Present	Present	Present
CTE stage	N/A	N/A	III	III	III	IV
Alzheimer’s disease	Absent	Absent	Absent	Absent	Absent	Absent
Lewy body disease	Transitional (limbic)	Absent	Absent	Absent	Absent	Absent
Frontotemporal lobar degeneration	Absent	Absent	Absent	Absent	Absent	Absent
Motor neuron disease	Absent	Absent	Absent	Present	Absent	Absent
ARTAG	Absent	Absent	Absent	Absent	Absent	Absent
TDP-43 in frontal cortex/MTL	Absent	Absent	Mild (frontal)	Mild (frontal)	Mild (frontal, hippocampus, entorhinal)	Mild (entorhinal)
CERAD neuritic amyloid plaque score	0	0	0	0	0	0
Diffuse Amyloid Plaque Score	0	Sparse	0	0	0	Sparse
Thal phase	0	1	0	0	0	1
Braak stage	0	0	IV	I	IV	III
Cause of death	Cardiovascular	Cancer	Cancer	Motor neuron disease	Cancer	Head/neck trauma from fall

Specific ages are not provided to protect confidentiality. Lower scores for the MoCA represent worse global cognitive status, whereas higher scores on the FAQ are representative of greater functional impairment

*ARTAG* aging-related tau astrogliopathy, *CTE* chronic traumatic encephalopathy, *FAQ* Functional Activities Questionnaire, *p-tau* hyper-phosphorylated tau, *MoCA* Montreal Cognitive Assessment, *SUVR* standard uptake value ratio

There are four evaluation sites for the DIAGNOSE CTE Research Project. For this study, one participant had PET scans at Boston University School of Medicine (with MRI conducted at Brigham and Women’s Hospital), two had PET scans at Cleveland Clinic Lou Ruvo Center for Brain Health in Las Vegas, two had PET scans at Mayo Clinic Arizona (with PETs conducted at Banner Alzheimer’s Institute), and one had PET scans at NYU Langone Medical Center. For the DIAGNOSE CTE Research Project, all sites received approval by their Institutional Review Boards and participants provided written informed consent. For the UNITE study, procedures have been approved by the BU Medical Campus and Bedford VA Hospital Institutional Review Boards. All next of kin or legal representatives of brain donors provided written informed consent.

### Imaging acquisition and analysis

MRIs across the four sites were conducted using the same 3 T MRI model (MAGNETOM Skyra; Siemens Healthineers, Erlangen, Germany). All images were acquired at high resolution (1 × 1 × 1 mm^3^, 176 slices, 256 × 256 cm^2^ field of view) in the sagittal plane using 3D sequences, including MPRAGE (repetition time (TR) = 2530 ms, echo time (TE) = 3.36 ms, inversion time (TI) = 1100 ms). One participant did not have an MRI (case 3). The PET-CT scanners were not identical across the four sites. The florbetapir protocol included a 370 MBq (10 mCi) bolus injection, immediately followed by acquisition of brain scans consisting of 10 frames, each one minute in length. Fifty minutes post-injection, the participant completed a second 15-min brain scan consisting of three frames, each of which required 5 min. PET images were reconstructed in a 128 × 128 matrix and a post hoc Gaussian filter = 5 mm. Corrections for random coincidences, scatter, system dead time, and attenuation were performed as provided by the camera manufacturer. Partial volume corrections were not performed because not all participants had an MRI scan. As described below, our goal was to match the PET ROIs to the tissue ROIs. To accomplish this, the ROIs were defined using various atlases which makes it challenging to perform partial volume correction consistently across all ROIs.

For the five participants with an MRI, reconstructed PET images were processed using PMOD software including motion correction and co-registration onto the participant’s MRI. The participant’s MRI was segmented into gray matter, white matter, and cerebrospinal fluid. Subsequently, the MRI was normalized into the standard MNI (Montreal Neurological Institute) and the same transformation was applied to the co-registered PET images. ROIs were defined by the standardized Automated Anatomic Labeling (AAL) volume of interest template. For the one participant without an MRI, the AAL template was applied to the PET data after it was normalized (there was no MRI co-registration). A positive florbetapir-PET scan was defined by a cortical composite SUVR score of 1.10 or greater (centiloid values > 24.3), corresponding to the presence of at least moderately frequent neuritic amyloid-β plaques in near end-of-life persons who agreed to brain donation prior to death [45].

The use of flortaucipir in this study was carried out through an Investigational New Drug (IND #131,391) from the U.S. FDA. All participants underwent flortaucipir PET scans after 370 MBq bolus injection (10 mCi). Five participants had dynamically acquired PET scans after 80 min post-injection for at least 20 min and one participant’s flortaucipir scan ended at 90 min after injection. Tracer doses were requested through Avid Radiopharmaceuticals (Philadelphia, PA, USA). Imaging calibration and quality control procedures were completed for all sites prior to study enrollment. Additional quality control including assessment for motion artifacts and ensuring that corrections were applied for randoms and scatter fraction was conducted on each scan by Invicro. The flortaucipir-PET scans were processed using a PET unified pipeline (PUP; https://github.com/ysu001/PUP) [46, 47]. For the five participants who had completed an MRI, the method included scanner harmonization filtering to reach a common 8-mm resolution [48], between-frame motion correction, frame summation, PET-to-MRI co-registration, and regional SUVR extraction based on the FreeSurfer generated anatomical regions of interest (ROIs) with bilateral cerebellar cortex as the initial reference region. FreeSurfer-processed T1-weighted MRI was spatially normalized using the Statistical Parametric Mapping (SPM) software, and the resulting warping fields were applied to the co-registered PET data to bring the PET into the MNI template space. For the one participant missing MRI data (case 3), the flortaucipir-PET data went through the first steps of PUP, scanner harmonization, motion correction, and summation. The summed flortaucipir-PET data were then transformed to the template space using a separate PET-only pipeline with a pre-established flortaucipir template from the whole cohort. Note that we did not apply a PET-only pipeline to all cases as co-registration of MRI is optimal when available. PET-to-PET template registration was carefully checked for the one participant without MRI, and there were no issues with registration.

Regions-of-interest (ROIs) were defined in MNI template space and were a priori selected based on their involvement in CTE [6–8], previous findings on flortaucipir distribution in living participants at risk for CTE [27, 29, 30], to mirror the neuropathological protocol described below, and/or are consistent with ROIs examined in flortaucipir-PET-pathological studies of AD [42]. Although the neuropathological protocol guided selection of the flortaucipir-PET ROIs, PET, and autopsy ROIs were not stereotactically matched, this is discussed as a limitation of the study. SUVRs from ROIs were re-normalized and derived using the cerebellum crus 1 as the final reference ROI. Note that p-tau can be found in the dentate nucleus of the cerebellum, but has not been reported in the cerebellum crus [8]. ROIs included the dorsolateral frontal cortex (DLFC), orbital frontal cortex (OFC), superior temporal cortex (STC), inferior parietal cortex (IPC), entorhinal cortex (EC), amygdala, hippocampus, and thalamus. Note that off-target flortaucipir binding has been described in the hippocampus due to spill-in effect from choroid plexus binding [49]. Off-target flortaucipir binding is also common in the thalamus [27, 30, 50]. Interpretation of associations for these regions are made with caution. Most of these regions are from the Automated Anatomical Labeling (AAL3) atlas [51]. The DLFC was defined in the Brodmann atlas provided by MRIcron and the EC was from the Mayo Clinic Adult Lifespan Template and Atlas (MCALT) [52]. Mean SUVRs were computed to form cortical (DLFC, OFC, ST, IP) and limbic (CA1-CA4, EC, amygdala) composites to be consistent with past research [42] and to reduce the number of analyses performed. SUVRs from the thalamic ROI were examined separately.

### Neuropathological evaluation

Neuropathological evaluations were performed by study neuropathologists and done blinded to clinical data. Neuropathological analyses and results are presented at a clinical-pathological consensus conference where at least one and typically two other study neuropathologists are present. Discrepancies or disagreements are resolved, and consensus is made for the final diagnoses. Three study neuropathologists (TS, BH, AM) evaluated the cases of the present study (TS, *n* = 3; BH, *n* = 1; AM, *n* = 2). Pathological processing and evaluation were conducted using published methodology [53, 54]. Brain weight and macroscopic features were recorded during initial processing. Twenty-two sections of paraffin-embedded tissue were stained for Luxol fast blue/hematoxylin and eosin (LHE), Bielschowsky’s silver, p-tau (AT8), alpha-synuclein, amyloid-beta (Aβ), and phosphorylated TDP-43 (pTDP-43) using methods described elsewhere [55]. Established criteria were used for the neuropathological diagnosis of neurodegenerative diseases [56–64]. The neuropathological diagnosis of CTE was made using criteria defined at two National Institute of Neurological Disorders and Stroke (NINDS) and National Institute of Biomedical Imaging and Bioengineering (NIBIB) sponsored consensus conferences [6, 7]. A CTE diagnosis required the presence of at least one pathognomonic perivascular neuronal p-tau lesion (astrocytic perivascular p-tau lesions were considered non-diagnostic in the absence of neuronal lesions) [6, 8, 13, 65]. CTE p-tau neuropathology was classified into four stages (stage IV being most severe) using the McKee staging criteria [8, 66]. Stage III and IV CTE are defined by diagnostic p-tau pathology in the cortex, with diffuse p-tau pathology extending into the medial temporal lobes, including the hippocampus, amygdala, and entorhinal cortex, diencephalon, and increased involvement of the brainstem. P-tau pathology is more widespread in stage IV compared to stage III CTE, often with neuronal loss and astrocytosis, and p-tau pathology involving the basis pontis and dentate nucleus of the cerebellum. Medial temporal lobe p-tau pathology in stage III and IV CTE is distinguished from primary age-related tauopathy (PART) by the predominant involvement of the CA4 and CA2 hippocampal subfields, clustered, patchy p-tau pathology in the amygdala, and striking superficial p-tau pathology in the entorhinal cortex, with prominent dotlike neurites [67].

Severity of regional p-tau was also rated using semi-quantitative scales (0 = none, 3 = severe). These scales were used to facilitate description of regional p-tau severity and not as primary outcomes. The three study neuropathologists have been shown to have excellent inter-rater reliability for CTE stage and the semi-quantitative ratings scales of p-tau severity (intra-rater reliability was not examined) [8].

Slides were digitized at × 20 magnification using an AT Turbo scanner (Leica Biosystems) and visualized with Aperio ImageScope (Leica Biosystem). The density of total AT8 staining was quantitatively measured in the DLFC, OFC, STC, IPC, EC, amygdala, hippocampus subfields CA1, CA2/CA3, and CA4, and the thalamus. These ROIs were selected for reasons mentioned previously (see “Imaging acquisition and analysis” section). Images of sampled histology regions have been shown elsewhere [7]. Slide scanning methods have been described elsewhere [8, 68]. The gray matter was highlighted from the pia to the boundary between the white and gray matter. Leica’s image analysis and automated counting software (Aperio positive pixel algorithm, version 9; Leica Biosystems) was calibrated for positive staining to detect AT8-immunoreactivity within the ROI. Counts were normalized to the area measured and presented as density of positively stained pixels within the analyzed region (positive pixels/mm^2^). For cortical regions, p-tau density was measured at the depth of the cortical sulcus (defined as the bottom third of two connecting gyri). As was done for the flortaucipir-PET ROIs, mean cortical (DLF, OFC, ST, IP) and limbic (CA1-CA4, EC, amygdala) composites were computed. The thalamus was examined separately.

### Participant characteristics

Antemortem data were acquired through the participants’ involvement in the DIAGNOSE CTE Research Project. Semi-structured interviews were performed, supplemented by online questionnaires, to collect data on demographics (e.g., age, education, race, and ethnicity); clinical, athletic, military, and TBI history; and other variables not relevant to the present study. The Montreal Cognitive Assessment (MoCA)[69] and Functional Activities Questionnaire (FAQ)[70] were used to clinically characterize the current sample, along with TES diagnostic status [15]. An aliquot of whole blood collected at the time of the baseline blood draw was used for *APOE* genotyping [43].

### Statistical analyses

Flortaucipir SUVR cutoffs for tau positivity in CTE do not exist and the sample size is insufficient to conduct the appropriate analyses to determine potential SUVR cutoff values in CTE. Qualitative assessments of flortaucipir-PET SUVRs and associated maps and p-tau aggregation at autopsy were performed. Spearman rho (*ρ*) correlation analyses tested the associations between flortaucipir SUVR and postmortem AT8 pathology for the mean cortical and limbic composites, as well as the thalamus. Post hoc analyses examined the individual regions that comprised the cortical and limbic composites. A *p* value less than 0.05 defined statistical significance. *P* values were false discovery rate (FDR) adjusted for the three primary analyses (i.e., cortical, limbic, thalamus). *P* values were not adjusted for the post hoc analyses that examined the individual regions comprising the composites. Importantly, the minimal detectable Spearman *ρ* coefficient is 0.91 based on an alpha of 0.05 and a sample size of six and 80% power. Therefore, given the small sample size and limited power, emphasis is placed on interpretation of effect sizes and based on the following guidelines: 0.0–0.19 very weak, 0.20–0.39 weak, 0.40–0.59 moderate, 0.60–0.79 strong, and 0.80–1.0 very strong [71].

## Results

### Participant characteristics

Participant characteristics are shown in Table 1. Participants in the sample were all men who self-identified as white. They included three former NFL players and three former college football players. On average, they were 59.00 (SD = 9.32) years of age and their PET scans were acquired 20.33 (SD = 13.08) months before their death. There were moderate to strong associations between age (at time of PET scan) and flortaucipir SUVRs and p-tau density at autopsy for the cortical composite (flortaucipir: *ρ* = 0.43, *p* = 0.40; p-density: *ρ* = 0.83, *p* = 0.04), limbic composite (flortaucipir: *ρ* = 0.71, *p* = 0.11; p-tau density: *ρ* = 0.66, *p* = 0.16), and the thalamic region (flortaucipir: *ρ* = 0.89, *p* = 0.02; p-tau density: *ρ* = 0.89, *p* = 0.02). Associations with PMI were generally weak or moderate: cortical composite (flortaucipir: *ρ* =  − 0.29, *p* = 0.58; p-tau density: *ρ* =  − 0.32, *p* = 0.54), limbic composite (flortaucipir: *ρ* =  − 0.64, *p* = 0.17; p-tau density: *ρ* =  − 0.23, *p* = 0.66), and thalamus (flortaucipir: *ρ* =  − 0.52, *p* = 0.29; p-tau density: *ρ* =  − 0.55, *p* = 0.26).

### Clinical status

Montreal Cognitive Assessment (MoCA) scores ranged from 20 to 28 and four had a score of 26 or lower. Four of the six participants met NINDS criteria for TES [15]. Of those with TES, one met criteria for “probable CTE” level of certainty, one met criteria for “possible CTE,” and two met criteria for “suggestive of CTE.” Of these four TES cases, two had TES cognitive impairment [15] with one having mild dementia (this participant had TES-probable CTE). The other three TES cases had minimal functional impairments. All TES cases also had neurobehavioral dysregulation. There were two who were *not* diagnosed with TES (cases 1 and 3) because they did not have a core clinical feature required for a TES diagnosis. Although both had cognitive concerns, they did not have neuropsychological impairment on tests of episodic memory or executive dysfunction. Neurobehavioral dysregulation was also not sufficiently present.

### Neuropathological findings

**Fig. 1:**
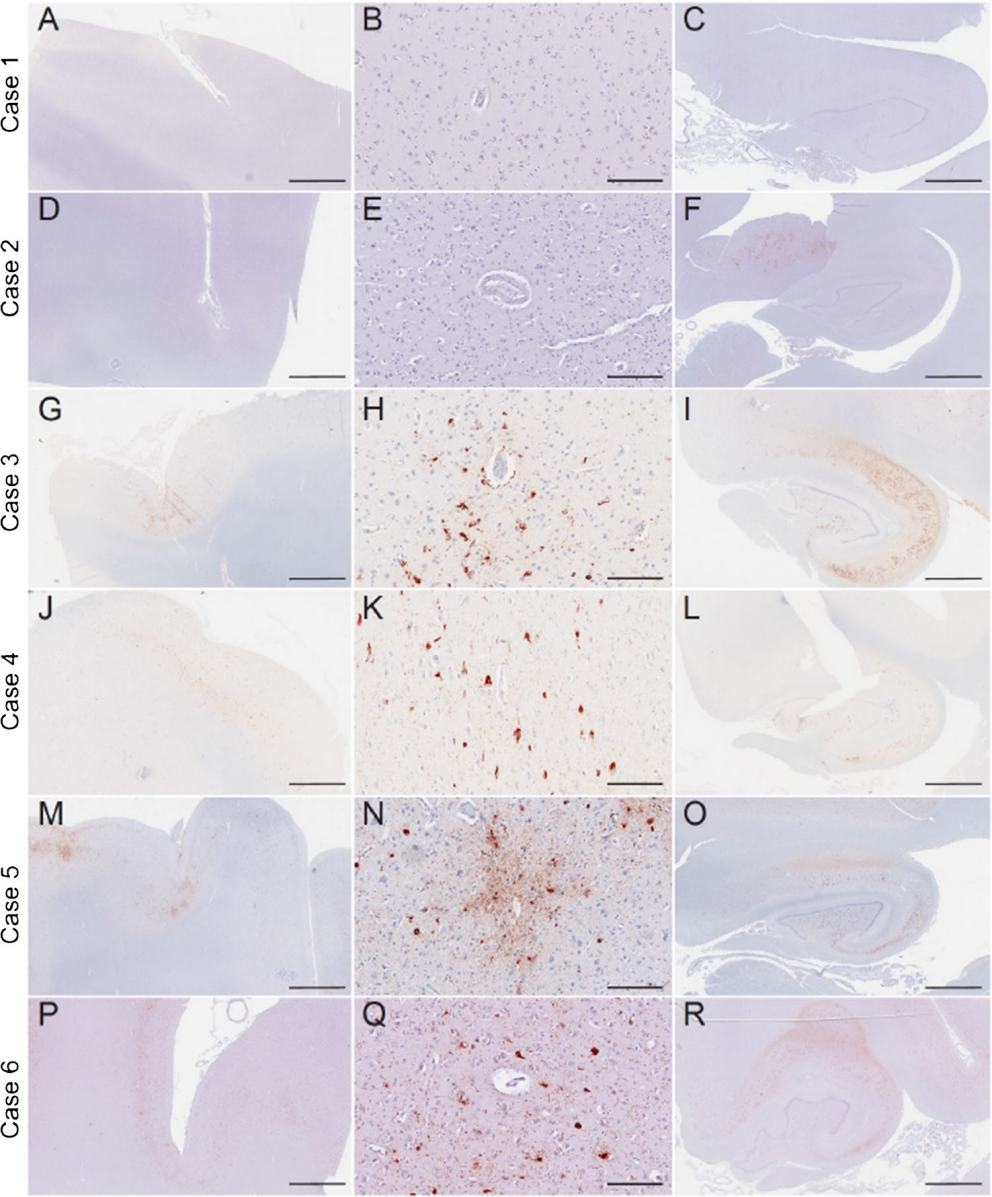
Phosphorylated tau neuropathology in the dorsolateral frontal cortex and hippocampus of six deceased American football players. Representative images of hyperphosphorylated tau (AT8 antibody) staining from former American football players. Of the six cases, two individuals did not receive a diagnosis of CTE (cases 1 and 2), three had CTE stage III (cases 3–5), and one had CTE stage IV (case 6). Of note, case 4 had low cortical tau burden (i.e., cortical sparring) but had high burden in the medial temporal lobes. The first column depicts a low power overview of cortical regions (A,D,G,J,M,P) (scale bar = 3 mm). All cortical images came from the dorsolateral frontal cortex except case 4, which came from the entorhinal cortex given it was a low cortical burden case of CTE. The second column shows a high-power view of perivascular tau pathology (B,E,H,K,N,Q) (scale bar = 200 µm). No perivascular tau was observed in cases 1 and 2. The third column depicts the posterior hippocampus (C,F,I,L,O,R) (scale bar = 3 mm)

Cases 1 and 2 did not meet neuropathological diagnostic criteria for CTE. Case 1 was neuropathologically diagnosed with limbic transitional Lewy body disease and case 2 had no neurodegenerative disease diagnosis. The remaining four cases had CTE (cases 3–6). Cases 3–5 had stage III CTE and case 6 had stage IV CTE (Fig. 1). Of the four CTE cases, one had motor neuron disease (case 4). None of the other CTE cases had co-morbid neurodegenerative disease diagnoses (e.g., AD, FTLD). All cases had a Braak score greater than 0. However, neuritic amyloid plaques were absent for all cases (CERAD = 0). Sparse diffuse amyloid plaques were present for two (cases 2 and 6) and absent in the other cases.

The two non-CTE cases (cases 1 and 2; both former college players) had the lowest overall burden of p-tau. None to minimal p-tau pathology was present across the cortical, limbic, and thalamic ROIs. Among the CTE cases, p-tau density was greatest in limbic regions, followed by the cortex. Among the cortical regions, p-tau density was greatest in the frontal cortices across all cases. There was moderate to severe involvement of the DLFC and STC three cases, case 4 had sparse cortical p-tau pathology. Case 5 also had moderate severity of p-tau of the OFC. Cases 5 and 6 had moderate IPC involvement, but p-tau pathology was otherwise absent or mild in the IPC across the cases. All CTE cases had moderate to severe involvement of the hippocampus, EC, and amygdala (case 5 had mild p-tau severity in the amygdala). Thalamus was the least affected; three cases had mild p-tau and case 3 had moderate p-tau pathology. Case 6 (stage IV CTE) had widespread p-tau pathology with severe involvement of the substantia nigra and p-tau in the dentate nucleus of the cerebellum (unaffected in the other CTE cases).

### Florbetapir PET

The participants’ florbetapir SUVRs are shown in Table 1. Five participants underwent florbetapir-PET; the sixth participant was scheduled, but there was a dose failure. All five PET scans were “amyloid-β negative,” consistent with CERAD absent-to-sparse neuritic amyloid-β plaques.

### Flortaucipir uptake

**Fig. 2:**
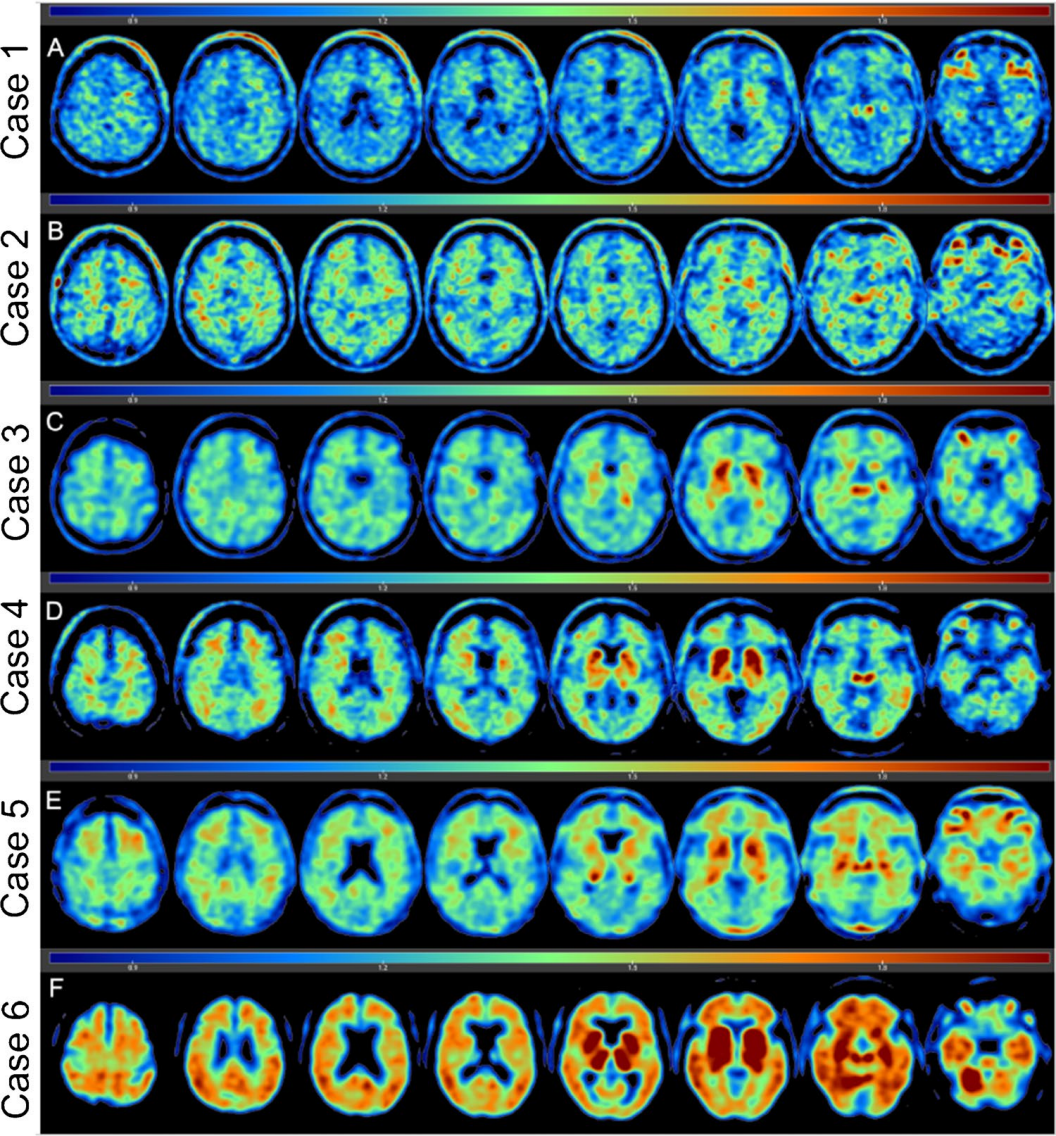
Flortaucipir PET images of six deceased American football players. Five participants had dynamically acquired PET scans after 80 min post-injection for at least 20 min and one participant’s flortaucipir scan ended at 90 min after injection. Voxel-wise SUVR values are represented relative to a cerebellar reference region and scaled for a range of 0–2.0. The flortaucipir PET images are of two former American football players without autopsy-confirmed CTE (a, b), three who had CTE stage III at autopsy (c–e), and one who had CTE stage IV at autopsy (f)

Flortaucipir SUVRs for the cortical composite, limbic composite, thalamic, and individual ROIs are shown in Table 2. Figure 2 shows flortaucipir SUVR maps for all six cases. Cases 5 and 6 had the highest SUVRs, followed by case 3. The remaining three cases had comparable SUVRs with cases 1 and 2 generally having the lowest, aligning with the no CTE diagnosis and sparse p-tau at autopsy. However, there was overlap in SUVRs between one of the CTE cases (i.e., case 4) and the two non-CTE cases. There was a consistent pattern of uptake for all cases. PET SUVRs were highest in limbic regions, particularly for the hippocampus, and in the thalamus. PET SUVRs were lowest in the cortical regions with greatest binding for the OFC, followed by the STC. Flortaucipir SUVRs for the DLFC were relatively similar across the cases. SUVRs were generally lowest in the IPC for the CTE cases.

**Table 2:** Antemortem flortaucipir PET SUVRs and postmortem p-tau density

	Case 1 (no CTE)	Case 2 (no CTE)	Case 3 (CTE stage III)	Case 4 (CTE stage III)	Case 5 (CTE stage III)	Case 6 (CTE stage IV)
Flortaucipir-PET SUVR						
Cortical	1.10	1.15	1.14	1.09	1.27	1.25
Frontal	1.10	1.14	1.17	1.08	1.33	1.27
Dorsolateral frontal	1.04	1.05	1.08	1.00	1.23	1.10
Orbital-frontal	1.16	1.22	1.26	1.16	1.43	1.44
Superior temporal	1.11	1.17	1.15	1.18	1.25	1.37
Inferior parietal	1.11	1.17	1.08	1.01	1.16	1.11
Limbic	1.18	1.27	1.30	1.23	1.55	1.69
Entorhinal	1.14	1.29	1.17	1.15	1.49	1.35
Amygdala	1.20	1.24	1.33	1.24	1.54	1.81
Hippocampus	1.21	1.30	1.40	1.30	1.63	1.89
Thalamus	1.20	1.35	1.41	1.49	1.46	1.89
AT8 p-tau density, positive pixels mm^2^						
Cortical	175.55	490.26	9141.35	571.16	17,045.71	16,233.65
Frontal	211.04	661.91	14,467.27	706.76	22,093.56	25,087.00
Dorsolateral frontal	241.50	426.22	27,765.76	736.59	30,128.24	47,963.93
Orbital-frontal	180.57	897.60	1168.77	676.93	14,058.87	2210.06
Superior temporal	182.31	318.33	6479.81	677.36	6831.73	2971.11
Inferior parietal	97.82	318.90	1151.05	193.77	17,164.00	11,789.51
Limbic	204.48	588.44	93,958.37	19,872.87	119,084.02	55,845.97
Entorhinal	181.10	1130.51	73,516.37	17,824.09	180,358.90	78,658.41
Amygdala	545.38	136.69	8059.85	3819.48	30,281.98	42,481.20
Hippocampus	98.64	558.34	129,405.21	25,906.93	128,259.73	52,696.75
CA1-Hippocampus	70.92	1172.90	210,210.79	18,072.52	103,603.49	92,860.40
CA2/3-Hippocampus	92.14	206.55	146,305.70	49,197.60	172,928.96	51,960.08
CA4-Hippocampus	132.85	295.57	31,699.15	10,450.67	108,246.75	13,269.76
Thalamus	157.61	348.82	1467.19	599.97	5185.09	9633.17

Cortical, frontal cortex, and limbic were mean composites that comprise these regions listed in the table. P-tau density was quantified using digitally scanned slides at × 20 magnification on a Leica Aperio ImageScope

*CTE* chronic traumatic encephalopathy, *p-tau* hyper-phosphorylated tau, *SUVR* standard uptake value ratio

### Correlations between flortaucipir PET and AT8 p-tau measurements

**Fig. 3:**
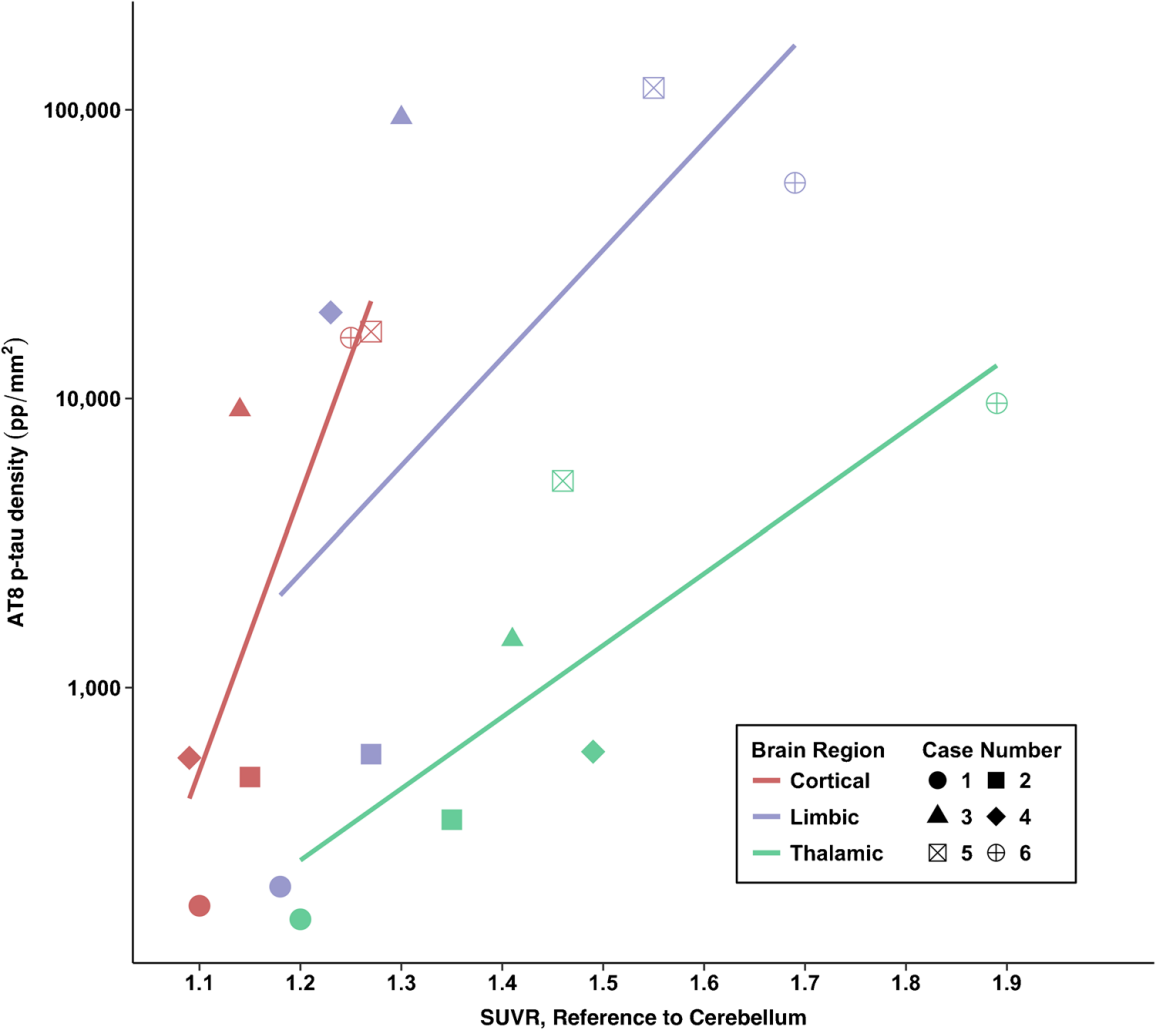
Associations between antemortem flortaucipir SUVRs and postmortem phosphorylated tau density. Cortical composite is the mean of the dorsolateral frontal cortex, orbital-frontal cortex, superior temporal cortex, and the inferior parietal cortex. Limbic composite is the mean of the entorhinal cortex, amygdala, and the hippocampus

**Fig. 4:**
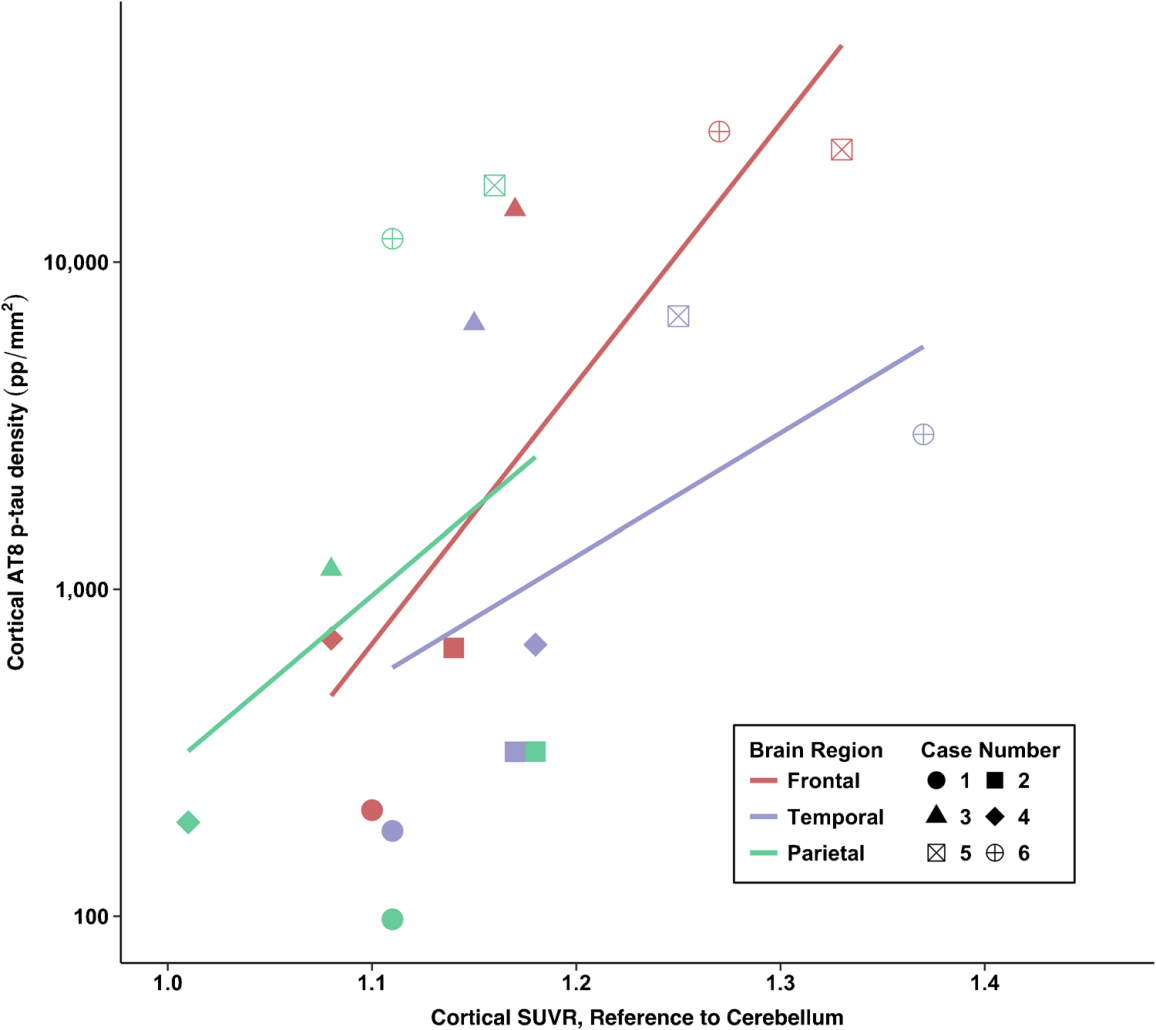
Association between antemortem flortaucipir SUVRs and postmortem phosphorylated tau density in cortical regions of interest

**Fig. 5:**
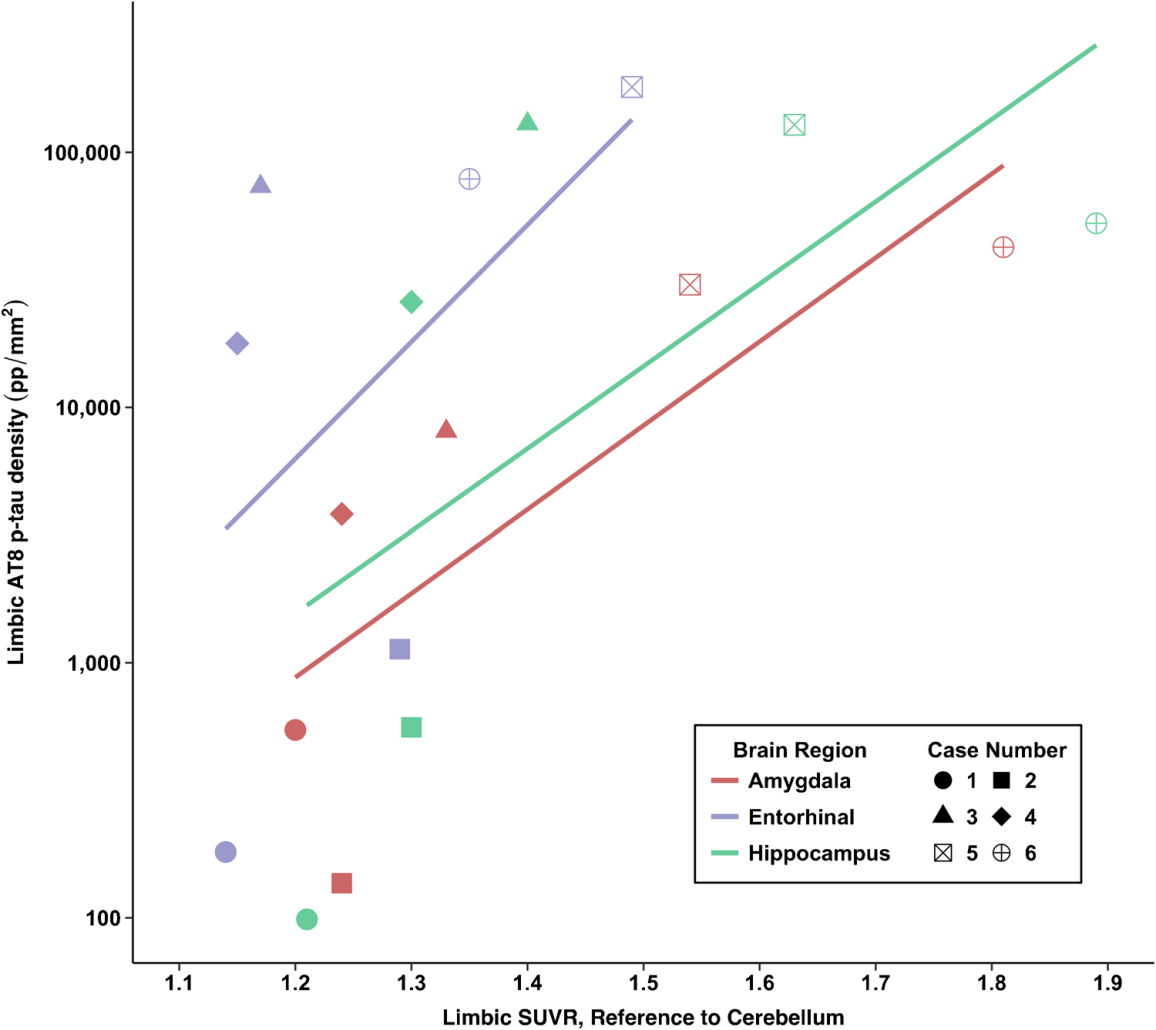
Association between antemortem flortaucipir SUVRs and postmortem phosphorylated tau density in limbic regions of interest

Figures 3, 4, and 5 show flortaucipir-AT8 associations. Across all regions except for the thalamus, p-tau densities at autopsy had a large dynamic range whereas there was a restricted range for flortaucipir SUVRs. For the thalamus, the opposite pattern was present. As described next, discrepancies between SUVRs and p-tau density at autopsy existed. There was a strong association between flortaucipir SUVR and postmortem p-tau density in the prespecified cortical (*ρ* = 0.71, FDR *p* value = 0.11) and limbic composites (*ρ* = 0.77, FDR *p* value = 0.11). The thalamus had the lowest p-tau density at autopsy but among the highest flortaucipir SUVRs. There was a very strong association between flortaucipir SUVR and p-tau density in the thalamus (*ρ* = 0.83, FDR *p* value = 0.13). When restricting the sample to those who had autopsy-confirmed CTE (*n* = 4), the flortaucipir–AT8 associations for the cortical composite were very strong (*ρ* = 1.00, *p* < 0.01). For the limbic composite, there was a moderate association (*ρ* = 0.40, *p* = 0.60). There was a moderate association for the thalamus (*ρ* = 0.40, *p* = 0.60). Two of the CTE cases had nearly identical thalamus flortaucipir SUVRs but discrepant p-tau density at autopsy, suggesting that the thalamic PET signal might be related to non-p-tau changes.

### *Post hoc*: correlations between flortaucipir PET and AT8 p-tau ROI measurements

There was a strong association between flortaucipir SUVR and postmortem p-tau density in the frontal composite (*ρ* = 0.77, *p* = 0.07). There were moderate associations for the STC (*ρ* = 0.54, *p* = 0.27) and IPC (*ρ* = 0.40, *p* = 0.40). For the STC, there were cases who had comparable SUVRs but discrepant p-tau density at autopsy (see cases 4 and 5 vs. case 2, Table 2 and Fig. 2). Cases 3 and 6 had the lowest and highest STC SUVRs among the CTE cases, respectively; however, this was not the case for p-tau density.

Of the limbic regions, there were very strong associations for the EC (*ρ* = 0.83, *p* = 0.04) and the amygdala (*ρ* = 0.83, *p* = 0.04). There was a strong association for the hippocampus (*ρ* = 0.77, *p* = 0.07). The two non-CTE cases had overlapping SUVRs of limbic regions relative to CTE cases but had the lowest p-tau density. For example, there were identical SUVRs for the hippocampus for case 2 (no CTE) and case 4 (CTE), but these two cases had substantially different p-tau density in this region. As previously mentioned, there is known off-target binding in the hippocampus due to spill-in effect from the choroid plexus.

## Discussion

This study compared near end-of-life flortaucipir PET to postmortem CTE-related p-tau pathology (defined by density of total AT8 staining from digital slide scanning) in six former American football players, including four who were diagnosed neuropathologically with CTE (stages III–IV) and two who did not meet criteria for CTE. There were strong associations between antemortem PET and postmortem neuropathological measurements of p-tau pathology in the prespecified composite cortical ROI (including the frontal cortex), limbic ROI (including the amygdala, EC and hippocampus), and thalamus. Although this limited case series does not include individuals with low stage CTE and does not clarify flortaucipir PET’s accuracy in the differential diagnosis of CTE (which is likely limited due to the modest SUVRs and overlap between clinically characterized groups), it suggests that flortaucipir PET may be useful for detecting high stage CTE neuropathology. Importantly, it also remains unclear if the flortaucipir signal is specifically detecting p-tau pathology or other neuropathological processes in CTE [72].

Mantyh et al. reported a non-significant and modest effect (*ρ* = 0.35) between antemortem flortaucipir-PET SUVRs and postmortem p-tau pathological burden in a single former NFL player who had autopsy-confirmed stage IV CTE [27]. Time from PET to death for that case was approximately 52 months. In this larger sample, the average interval from PET to autopsy was approximately 20 months. The frontotemporal distribution of flortaucipir uptake in this sample is consistent with Mantyh et al.[27] and previous flortaucipir-PET imaging studies among living individuals at high risk for CTE [29, 30]. This pattern of uptake mimics the cortical distribution of p-tau in CTE [8], including that observed in this sample. There was also a high concordance between flortaucipir uptake and cortical p-tau density, particularly for the frontal cortex. Associations for the STC and IPC were weaker and more variable, and CTE and non-CTE cases had overlapping SUVRs but discrepant p-tau density at autopsy. Some of this variability could have been driven by case 4 who had minimal, yet diagnostic, cortical p-tau pathology.

CTE stages III and IV are characterized by p-tau pathology in the hippocampus, EC, and amygdala [8]. Here, there was a modest association between flortaucipir and p-tau density for the limbic composite in those with CTE. Flortaucipir SUVRs were greatest in the hippocampus but had relatively modest association with hippocampal p-tau density. There were cases with similar SUVRs and different levels of p-tau in the hippocampus. Notably, CTE and non-CTE cases had identical SUVRs in the hippocampus but discordant p-tau density at autopsy. This pattern supports non-tau-related binding and raises concern for the diagnostic usefulness of flortaucipir to detect CTE p-tau pathology in the hippocampus. Off-target flortaucipir binding has been described in the hippocampus due to spill-in effect from choroid plexus binding [49]. Off-target flortaucipir binding is also common in the thalamus and basal ganglia [27, 30, 50]. The thalamus had among the highest flortaucipir SUVRs and the lowest p-tau density at autopsy. Although there might be off-target flortaucipir binding in the hippocampus and the thalamus, these regions are severely affected in high stage CTE[8] and can have molecular and neurodegenerative changes from exposure to RHI [30, 73–77]. Although speculative, the high flortaucipir uptake in the hippocampus and thalamus might be capturing non-tau neuropathological changes associated with CTE and/or exposure to RHI that co-localize with p-tau. Given that co-morbid neurodegenerative diseases were absent in the four CTE cases, the pathologies being captured could be non-specific neuropathological changes associated with general neurodegenerative changes [50, 78–80]. Of note, elevated flortaucipir signal has been described in atrophic regions in patients with autopsy-proven, tau-negative neurodegeneration [72]. The known off-target flortaucipir binding in the hippocampus and thalamus limits interpretation of observed associations.

There were also associations between flortaucipir SUVR and p-tau density in the EC and amygdala. Of the limbic regions, off-target flortaucipir binding predominantly affects measurement of the hippocampus [49]. The EC and amygdala are severely affected in high stage CTE [8]. ARTAG and PART are other tauopathies characterized by deposition of p-tau in the MTL that are nearly universal with increased age. While the distribution and nature of the limbic p-tau pathology was neuropathologically interpreted as CTE in the four cases, it is impossible to exclude contribution of PART to the limbic neurofibrillary pathology[67] and to the flortaucipir SUVRs. A review of the neuropathological distinctions between CTE, ARTAG, and PART is provided elsewhere [13].

Flortaucipir SUVRs in this sample were similar to previous studies in CTE[27, 29, 30] and modest and lower than those reported in AD [39, 81]. For example, in patients with AD dementia, flortaucipir SUVRs have been shown to be 1.73 in the entorhinal cortex and 2.09 in the inferior temporal cortex [39]. (Note: direct comparison of SUVRs across studies can be difficult due to variations in reference region chosen.) In vivo studies also demonstrate small effect sizes for differences in flortaucipir SUVRs between participants at high risk for CTE and control groups [29, 30]. The flortaucipir SUVRs observed in CTE are more consistent with those in non-AD neurodegenerative diseases [38, 39, 72, 81]. Flortaucipir was developed to bind to the 3R/4R tau isoforms in AD[31–33] and might have better binding affinity to 3R tau [37, 38, 82]. Flortaucipir has limited specific binding to 4R isoforms of other tauopathies, such as progressive supranuclear palsy and corticobasal degeneration [39, 72, 82, 83]. Although CTE is a mixed 3R/4R tauopathy, the CTE tau isoforms might shift from 4 to 3R in later disease stages and binding affinity might vary by disease stage [9]. This could explain the increased concordance between antemortem flortaucipir and later stage p-tau density in this study. The molecular structure of p-tau in CTE is also distinct from AD and other tauopathies [10, 11]. In the context of modest binding affinity, the clinical meaning of the flortaucipir uptake and p-tau deposition in this sample is also unclear and there were discrepancies between diagnoses of TES, flortaucipir uptake, and CTE presence. This is the first clinicopathological correlation study with the 2021 TES research diagnostic criteria and there was misclassification of two cases. Due to the small sample size, we restricted our analyses to test the primary objectives of the study and thus did not formally test associations with clinical data, including TES diagnoses. However, flortaucipir and clinical associations will be tested using the larger DIAGNOSE CTE Research Project sample. Brain donation is also ongoing for this study and will allow for larger clinical-pathological correlation studies in the future.

The FDA-approved flortaucipir (or TAUVID) to estimate density and distribution of aggregated tau NFTs in older adults with cognitive impairment being evaluated for AD. It is noteworthy that the single limitation of use included in the TAUVID FDA prescribing information states: “TAUVID is not indicated for use in the evaluation of patients for chronic traumatic encephalopathy (CTE).” While there are limitations of clinical utility of flortaucipir in CTE, it could still offer differential diagnostic information relevant to the presence of AD; this remains to be determined in larger samples with disease comparison groups. There is a need for the development of radiotracer compounds with high affinity to the specific tau isoforms of CTE that would detect p-tau in early disease stages (e.g., CTE stage I or II). This may prove challenging given that p-tau aggregates in early-stage CTE tend to be isolated and patchy epicenters that are located at the depths of cortical sulci. Second-generation PET radiotracers (e.g., MK-6240, PI-2620, APN-1607) with less off-target binding and/or possible 4R binding and improved pharmacokinetics are currently under investigation to determine their usefulness as a biomarker of CTE [84]. However, these were also developed to detect tau associated with AD and may therefore be less applicable to CTE.

The present findings have limitations. The sample size is small and therefore has limited statistical power, generalizability, and the ability to account for potential confounding factors. The small sample size also precluded the ability to test the diagnostic accuracy of flortaucipir PET in CTE; it is an important target for future larger PET-to-autopsy studies in CTE. The interval between PET and post mortem, as well as the different PET scanners used across sites, might have contributed to variability in correlation between SUVR and AT8. Partial volume correction was also not performed for reasons described and this might have influenced estimation of tau PET measurements, but it is likely to have been an underestimation. The sample was composed of only white men and inferences to other populations cannot be made. Off-target binding in the hippocampus might have been exacerbated if blacks or African Americans were included in the sample given flortaucipir SUVRs are higher in the choroid plexus (and perhaps in the vicinity of leptomeninges) in blacks compared with whites due to off-target binding to melanin [49]. Attention to black or African American football players is important for future studies. PET imaging is costly, not reimbursed by health insurance, and not accessible within low- or middle-income sectors, thereby limiting its clinical use. The neuropathological protocol guided the selection of PET ROIs that were chosen based on regions most affected in CTE. Because regions were not stereotactically matched, discrepancy in the precise location that were analyzed across the PET and neuropathology protocols might have affected the associations between flortaucipir SUVRs and p-tau density at autopsy. The sample did not include brain donors with low stage CTE. To fully understand how the flortaucipir tracer behaves as a biomarker across the disease continuum, it will be important to conduct PET-to-autopsy studies among a larger sample of individuals who have CTE across the disease continuum. Finally, there was absence of brain donors with a history of RHI as well as disease comparison groups (e.g., AD).

## Conclusions

Findings from this PET-to-autopsy case series of six deceased former American football players suggest that flortaucipir PET may be useful for detecting high stage CTE neuropathology. There remains a need to develop and validate in vivo biomarkers that can detect the specific p-tau species of CTE across the disease continuum, including in early-stage disease.

## Data Availability

The datasets generated during and/or analyzed during the current study are available from the corresponding author on reasonable request.
